# Multi-Focus Fusion Technique on Low-Cost Camera Images for Canola Phenotyping

**DOI:** 10.3390/s18061887

**Published:** 2018-06-08

**Authors:** Thang Cao, Anh Dinh, Khan A. Wahid, Karim Panjvani, Sally Vail

**Affiliations:** 1 Department of Electrical and Computer Engineering, University of Saskatchewan, Saskatoon, SK S7N 5A9, Canada; thang.cao@usask.ca (T.C.); khan.wahid@usask.ca (K.A.W.); karim.panjvani@usask.ca (K.P.); 2 Agriculture and Agri-Food Canada, Ottawa, ON K1A 0C5, Canada; Sally.Vail@agr.gc.ca

**Keywords:** image fusion, multi-focus, weight maps, gradient domain, fast guided filter.

## Abstract

To meet the high demand for supporting and accelerating progress in the breeding of novel traits, plant scientists and breeders have to measure a large number of plants and their characteristics accurately. Imaging methodologies are being deployed to acquire data for quantitative studies of complex traits. Images are not always good quality, in particular, they are obtained from the field. Image fusion techniques can be helpful for plant breeders with more comfortable access plant characteristics by improving the definition and resolution of color images. In this work, the multi-focus images were loaded and then the similarity of visual saliency, gradient, and color distortion were measured to obtain weight maps. The maps were refined by a modified guided filter before the images were reconstructed. Canola images were obtained by a custom built mobile platform for field phenotyping and were used for testing in public databases. The proposed method was also tested against the five common image fusion methods in terms of quality and speed. Experimental results show good re-constructed images subjectively and objectively performed by the proposed technique. The findings contribute to a new multi-focus image fusion that exhibits a competitive performance and outperforms some other state-of-the-art methods based on the visual saliency maps and gradient domain fast guided filter. The proposed fusing technique can be extended to other fields, such as remote sensing and medical image fusion applications.

## 1. Introduction

The sharp increase in demand for global food raises the awareness of the public, especially agricultural scientists, to global food security. To meet the high demand for food in 2050, agriculture will need to produce almost 50 percent more food than was produced in 2012 [1]. There are many ways to improve yields for canola and other crops. One of the solutions is to increase breeding efficiency. In the past decade, advances in genetic technologies, such as next generation DNA sequencing, have provided new methods to improve plant breeding techniques. However, the lack of knowledge of phenotyping capabilities limits the ability to analyze the genetics of quantitative traits related to plant growth, crop yield, and adaptation to stress [2]. Phenotyping creates opportunities not only for functional research on genes, but also for the development of new crops with beneficial features. Image-based phenotyping methods are those integrated approaches that enable the potential to greatly enhance plant researchers’ ability to characterize many different traits of plants. Modern advanced imaging methods provide high-resolution images and enable the visualization of multi-dimensional data. The basics of image processing have been thoroughly studied and published. Readers can find useful information on image fusion in the textbooks by Starck or Florack [3,4]. These methods allow plant breeders and researchers to obtain exact data, speed up image analysis, bring high throughput and high dimensional phenotype data for modeling, and estimate plant growth and structural development during the plant life cycle. However, with low-cost and low-resolution sensors, phenotyping would meet some obstacles to solve low-resolution images. To cope with this issue, image fusion is a valuable choice.

Image fusion is a technique that combines many different images to generate a fused image with highly informative and reliable information. There are several image fusion types, such as multi-modal and multi-sensor image fusion. In multi-modal image fusion, two different kinds of images are fused, for example, combining a high-resolution image with a high-color image. In multi-sensor image fusion, images from different types of sensors are combined, for example, combining an image from a depth sensor with an image from a digital sensor. Image fusion methods can be divided into three levels depending on the processing: pixel level, feature level, and decision level [5,6]. Image fusion at the pixel level refers to an imaging process that occurs in the pixel-by-pixel manner in which each new pixel of the fused image obtains a new value. At a higher level than the pixel level, feature-level image fusion first extracts the relevant key features from each of the source images and then combines them for image-classification purposes such as edge detection. Decision-level image fusion (also named as interpretation-level or symbol-level image fusion) is the highest level of image fusion. Decision-level image fusion refers to a type of fusion in which the decision is made based on the information separately extracted from several image sources.

Over the last two decades, image fusion techniques have been widely applied in many areas: medicine, mathematics, physics, and engineering. In plant science, many image fusion techniques are being deployed to improve the classification accuracy for determining plant features, detecting plant diseases, and measuring crop diversification. Fan et al. [7] well implemented a Kalman filtering fusion to improve the accuracy of the prediction on the citrus maturity. In a related work, a feature-level fusion technique [8] was successfully developed to detect some types of leaf disease with excellent results. In other similar research, apple fruit diseases were detected by using feature-level fusion in which two or more color and feature textures were combined [9]. Decision-level fusion techniques have been deployed to detect crop contamination and plague [10]. Dimov et al. [11] have also implemented the Ehler’s fusion algorithm (decision level) to measure the diversification of the three critical crop systems with the highest classification accuracy. These findings suggest that image-fusion techniques at many levels are broadly applied in the plant science sector because they offer the highest classification accuracy.

Currently, many multi-focus image fusion techniques have been developed [12,13]. The techniques can be categorized into two classes: spatial domain methods and frequency domain methods. In the spatial domain methods, source images are directly fused, in which the information of image pixels are directly used without any pre-processing or post-processing. In the frequency domain methods, source images are transformed into frequency domain, and then combined. Therefore, frequency domain methods are more complicated and time-consuming than spatial domain methods. Many studies investigated multi-focus image fusion techniques in spatial and frequency domains to improve the outcomes. Wan et al. [14] proposed a method based on the robust principal component analysis in the spatial domain. They developed this method to form a robust fusion technique to distinguish focused and defocused areas. The method outperforms wavelet-based fusion methods and provides better visual perception; however, it has a high computation cost. In the similar spatial domain, a multi-focus image fusion method based on region [15] was developed, in which, their algorithm offers smaller distortion and a better reflection of the edge information and importance of the source image. Similarly, Liu et al. [16] investigated a fusion technique based on dense scale invariant feature transform (SIFT) in the spatial domain. The method performs better than other techniques in terms of visual perception and performance evaluation, but it requires a high amount of memory. In the frequency domain, Phamila and Amutha [17] conducted a method based on Discrete Cosine Transform. The process computes and obtains the highest variance of the 8 × 8 DCT coefficients to reconstruct the fused image. However, the method suffers from undesirable side effects such as blurring and artifact. In a recently published article, the authors reviewed the works using sparse representation (SR)-based methods on multi-sensor systems [18]. Based on sparse representation, the same authors also developed the image fusing method for multi-focus and multi-modality images [19]. This SR method learns an over-complete dictionary from a set of training images for image fusion, it may result in a huge increment of computational complexity.

To deal with these obstacles mentioned above, a new multi-focus image fusion based on the image quality assessment (IQA) metrics is proposed in this paper. The proposed fusion method is developed based on crucial IQA metrics and a gradient domain fast guided image filter (GDFGIF). This approach is motivated by the fact that visual saliency maps, including visual saliency, gradient similarity, and chrominance similarity maps, outperform most of the state-of-the-art IQA metrics in terms of the prediction accuracy [20]. According to Reference [20], visual saliency similarity, gradient similarity, and chrominance maps are vital metrics in accounting for the visual quality of image fusion techniques. In most cases, changes of visual saliency (VS) map can be a good indicator of distortion degrees and thus, VS map is used as a local weight map. However, VS map does not work well for the distortion type of contrast change. Fortunately, the image gradient can be used as an additional feature to compensate for the lack of contrast sensitivity of the VS map. In addition, VS map does not work well for the distortion type change of color saturation. This color distortion cannot be well represented by gradient either since usually the gradient is computed from the luminance channel of images. To deal with this color distortion, two chrominance channels are used as features to represent the quality degradation caused by color distortion. These IQA metrics have been proved to be stable and have the best performance [20]. In addition, gradient domain guided filter (GDGIF) [21] and fast guided filter (FGF) [22] are adopted in this work as the combination of GDGIF and FGF and can offer fast and better fused results, especially near the edges, where halo artifacts appear in the original guided image filter. This study focuses on how to fuse multi-focus color images to enhance the resolution and quality of the fused image using a low-cost camera. The proposed image fusion method was developed and compared with other state-of-the-art image fusion methods. In the proposed multi-focus image fusion, two or more images captured by the same sensor from the same visual angle but with a different focus are combined to obtain a more informative image. For example, a fused image with clearer canola seedpods can be produced by fusing many different images of a canola plant acquired by the same Pi camera at the same angle with many different focus lengths.

## 2. Methodology

### 2.1. Data Acquisition System

**Figure 1:**
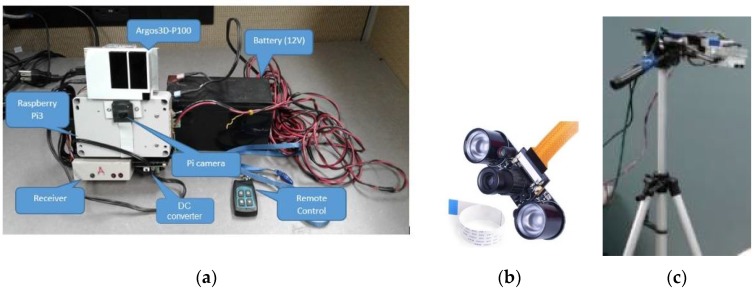
(**a**) Low-cost mobile phenotyping system; (**b**) Adjustable focus Pi camera; (**c**) System mounted on a tripod looking down to the canola plants.

This image fusion work is part of the development of a low-cost, high throughput phenotyping mobile system for canola in which a low-cost Raspberry Pi camera is used as a source of image acquisition. This system includes a 3D Time-of-Flight camera, a Pi camera, a Raspberry Pi3 (RP3), and appropriate power supplies for the cameras and the mini computer (Raspberry Pi3). A built-in remote control allows the user to start and stop image recording as desired. Figure 1 shows various components of the data acquisition system. Data are recorded in the SD card of the RP3 and retrieved using USB connection to a laptop before the images are processed. The Kuman for Raspberry Pi 3 Camera Module with adjustable focus is used in this system. This camera is connected to the Raspberry Pi using the dedicated CSI interface. The Pi camera equips to the 5 megapixels OV5647 sensor. It is capable of capturing 2592 × 1944 pixels static images; it also supports video capturing of 1080 p at 30 fps, 720 p at 60 fps, and 640 × 480 p at 60/90 formats.

The testing subjects were the canola plants at different growing stages. The plants were growing in a controlled environment and also in the field. To capture images of the canola, the plants were directly placed underneath the Pi camera that fixed on the tripod at a distance of 1000 mm (Figure 1). Each canola plant was recorded at 10 fps for 3 s. The time between each change of the focal length is 10 s. Only frame number 20 of each video stream acquired from the Pi camera was extracted to store in the database for later use. The reason for selecting the 20th frame is that the plants and the camera are required to be stable before the images are being captured and processed. Only the regions containing the plant in the selected images were cropped and used for multi-focus image fusion methods.

### 2.2. Image Fusion Algorithm

**Figure 2:**
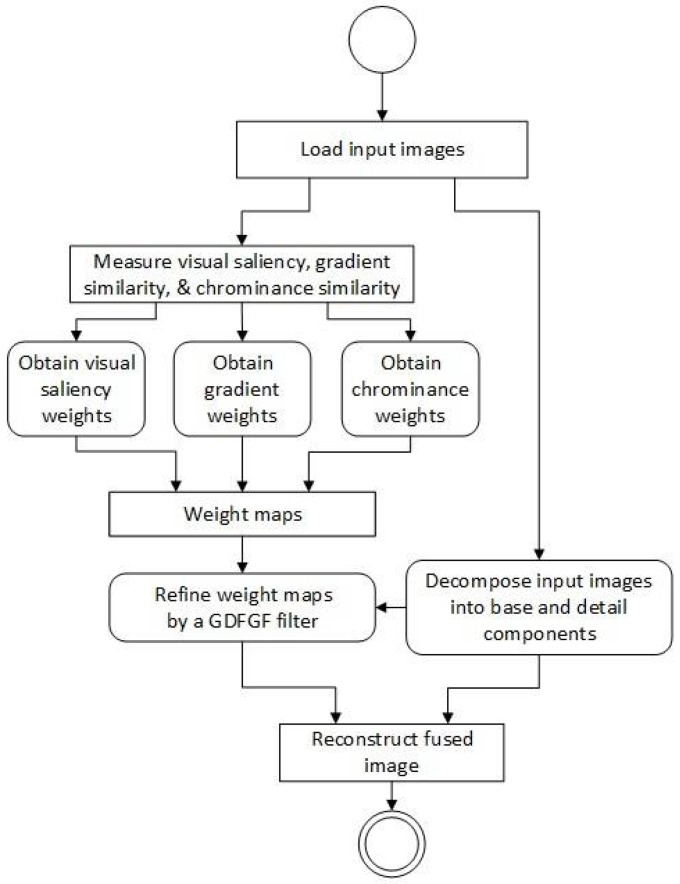
Workflow of the proposed multi-focus image fusion algorithm.

In the proposed fusion approach, three image quality assessment (IQA) metrics: visual saliency similarity, gradient similarity, and chrominance similarity (or color distortion) are measured to obtain their weight maps. Then these weight maps are refined by a gradient domain fast guided filter in which, a gradient domain guided filter proposed by Reference [21] and a fast guided filter proposed by Reference [22] are combined. The workflow of the proposed multi-focus image fusion algorithm is illustrated in Figure 2. The detail of the proposed algorithm is described as follows.

First, each input image is decomposed into a base and detailed component, which contain the large-scale and small-scale variations in intensity. A Gaussian filter is used for each source image to obtain its base component, and the detailed component can be easily obtained by subtracting the base component from the input image, as given by:

(1) $$B_{n}=I_{n}*G_{r,\sigma }$$

(2) $$D_{n}=I_{n}-B_{n}$$

where $B_{n}$ and $D_{n}$ are the base and detail component of the $n^{th}$ input image, respectively. ∗ denotes convolution operator, and $G_{r,\sigma }$ is a 2-D Gaussian smoothing filter.

Several measures were used to obtain weight maps for image fusing. Visual saliency similarity, gradient similarity, and chrominance maps are vital metrics in accounting for the visual quality of image fusion techniques [20]. In most cases, changes of visual saliency (VS) map can be a good indicator of distortion degrees and thus, VS map is used as a local weight map. However, VS map does not work very well for the distortion type of contrast change. Fortunately, the gradient modulus can be used as an additional feature to compensate for the lack of contrast sensitivity of the VS map. In addition, VS map does not work well for the distortion type change of color saturation. This color distortion cannot be well represented by gradient either since usually gradient is computed from the luminance channel of images. To deal with this color distortion, two chrominance channels are used as features to represent the quality degradation caused by color distortion. Motivated by these metrics, an image fusion method is designed based on the measurement of the three key visual features of input images.

#### 2.2.1. Visual Saliency Similarity Maps

A saliency similarity detection algorithm proposed by [23] is adopted to calculate visual saliency similarity due to its higher accuracy and low computational complexity. This algorithm is constructed by combining three simple priors: frequency, color, and location. The visual saliency similarity maps are calculated as

(3) $$VS_{n}^{k}=SF_{n}^{k}\cdot SC_{n}^{k}\cdot SD_{n}^{k}$$

where $SF_{n}^{k}$, $SC_{n}^{k}$, $SD_{n}^{k}$ are the saliency at pixel k under frequency, color and location priors. $SF_{n}^{k}$ is calculated by

(4) $$SF_{n}^{k}={\left(IL_{n}^{k}*g\right)}^{2}+{\left(Ia_{n}^{k}*g\right)}^{2}+{\left(Ib_{n}^{k}*g\right)}^{2}{)}^{1/2}$$

where $IL_{n}^{k}$, $Ia_{n}^{k}$, $Ib_{n}^{k}$ are three resulting channels transformed from the given RGB input image, $I_{n}$ to CIEL*a*b* space. * denotes the convolution operation. CIEL*a*b* is an opponent color system that a* channel represents green-red information while b* channel represents blue-yellow information. If a pixel has a smaller (greater) a* value, it would seem greenish (reddish). If a pixel has a smaller (greater) b* value, it would seem blueish (yellowish). Then, if a pixel has a higher a* or b* value, it would seem warmer; otherwise, colder. The color saliency $SC_{n}$ at pixel k is calculated using

(5) $$SC_{n}^{k}=1-\exp\left(-\frac{{\left(Ia_{n}^{k}\right)}_{}^{2}+{\left(Ib_{n}^{k}\right)}_{}^{2}}{\sigma_{C}^{2}}\right)$$

where $\sigma_{C}$ is a parameter. $Ia_{n}^{k}=\frac{Ia_{n}^{k}-mina}{maxa-mina}$, $Ib_{n}^{k}=\frac{Ib_{n}^{k}-minb}{maxb-minb}$, mina(maxa) is the minimum (maximum) value of the Ia and *minb* (*maxb*) is the minimum (maximum) value of the Ib.

Many studies found that the regions near the image center are more attractive to human visual perception [23]. It can thus be suggested that regions near the center of the image will be more likely to be “salient” than the ones far away from the center. The location saliency at pixel *k* under the location prior can be formulated by

(6) $$SD_{n}^{k}=\exp\left(-\frac{{\left\|k-c\right\|}^{2}}{\sigma_{D}^{2}}\right)$$

where $\sigma_{D}$ is a parameter. *c* is the center of the input image $I_{n}$. Then, the visual saliency is used to construct the visual saliency (VS) maps, given by

(7) $$VSm=VS*G_{r,\sigma }$$

where $G_{r,\sigma }$ is a Gaussian filter.

#### 2.2.2. Gradient Magnitude Similarity

According to Zhang et al. [24], the gradient magnitude is calculated as the root mean square of image directional gradients along two orthogonal directions. The gradient is usually computed by convolving an image with a linear filter such as the classic Sobel, Prewitt and Scharr filters. The gradient magnitude similarity algorithm proposed by Reference [24] is adopted in this study. This algorithm uses a Scharr gradient operator, which could achieve slightly better performance than Sobel and Prewitt operators [25]. With the Scharr gradient operator, the partial derivatives $GMx_{n}^{k}$ and $GMy_{n}^{k}$ of an input image $I_{n}$ are calculated as:

(8) $$GMx_{n}^{k}=\frac{1}{16}\left[\begin{array}{ccc} 3 & 0 & -3\\ 10 & 0 & -10\\ 3 & 0 & -3 \end{array}\right]*I_{n}^{k}\;GMy_{n}^{k}=\frac{1}{16}\left[\begin{array}{ccc} 3 & 0 & -3\\ 10 & 0 & -10\\ 3 & 0 & -3 \end{array}\right]*I_{n}^{k}$$

The gradient modulus of the image $I_{n}$ is calculated by

(9) $$GM_{n}=\sqrt{GMx_{}^{2}+GMy_{}^{2}}$$

The gradient is computed from the luminance channel of input images that will be introduced in the next section. Similar to the visual saliency maps, the gradient magnitude (GM) maps is constructed as

(10) $$GMm=GM*G_{r,\sigma }$$

#### 2.2.3. Chrominance Similarity 

The RGB input images are transformed into an opponent color space, given by

(11) $$\left[\begin{array}{c} L\\ M\\ N \end{array}\right]=\left[\begin{array}{ccc} 0.06 & 0.63 & 0.27\\ 0.30 & 0.04 & -0.35\\ 0.34 & -0.6 & 0.17 \end{array}\right]\left[\begin{array}{c} R\\ G\\ B \end{array}\right]$$

The *L* channel is used to compute the gradients introduced in the previous section. The *M* and *N* (chrominance) channels are used to calculate the color distortion saliency, given by

(12) $$M_{n}=0.30*R+0.04*G-0.35*B$$

(13) $$N_{n}=0.34*R-0.6*G+0.17*B$$

(14) $$C_{n}=M_{n}\cdot N_{n}$$

Finally, the chrominance similarity or color distortion saliency (CD) maps are calculated by

(15) $$CDm=C*G_{r,\sigma }$$

#### 2.2.4. Weight Maps 

Using three measured metrics above, the weight maps are computed as given by

(16) $$W_{n}={\left(VSm\right)}^{\alpha }\cdot {\left(GMm\right)}^{\beta }\cdot {\left(CDm\right)}^{ɤ}$$

where α, β, and ɤ are parameters used to control the relative importance of visual saliency (VS), gradient saliency (GM), and color distortion saliency (CD). From these weight maps, W at each location *k*, the overall weight maps of each input image can be obtained.

(17) $$W_{n}^{k}=\{\begin{array}{ll} 1, & if\;W_{n}^{k}=\max\left(W_{1}^{k},\;W_{2}^{k},\ldots ,W_{N}^{k}\right),\\ 0, & otherwise, \end{array}$$

where *N* is the number of input images, $W_{n}^{k}$ is the weight value of the pixel k in the $n^{th}$ image. Then proposed weight maps are determined by normalizing the saliency maps as follows:

(18) $$W_{n}^{k}=\frac{W_{n}^{k}}{\sum_{n=1}^{N}W_{n}^{k}},\;\forall n=1,2,\ldots ,N$$

These weight maps are then refined by a gradient domain guided filter described in the next section.

#### 2.2.5. Gradient Domain Fast Guided Filter 

The gradient domain guided filter proposed by Reference [21] is adopted to optimize the initial weight maps. By using this filter, the halo artifacts can be more effectively suppressed. It is also less sensitive to its parameters but still has the same complexity as the guided filter. The gradient domain guided filter has good edge-preserving smoothing properties as the bilateral filter, but it does not suffer from the gradient reversal artifacts. The filtering output is a local linear model of the guidance image. This is one of the fastest edge-preserving filters. Therefore, the gradient domain guided filter can apply in image smoothing to avoid ringing artifacts.

It is assumed that the filtering output Q is a linear transform of the guidance image G in a local window $w_{k}$ centered at pixel k.

(19) $$Q_{i}=a_{k}G_{i}+b_{k},\;\forall i\in w_{k}$$

where $\left(a_{k},\;b_{k}\right)$ are some linear coefficients assumed to be constant in the local window $w_{k}$ with the size of (2ζ1+1)×(2ζ1+1). The linear coefficients ($a_{k},\;b_{k})$ can be estimated by minimizing the cost function in the window $w_{k}$ between the output image *Q* and the input image *P*

(20) $$E_{\left(a_{k},b_{k}\right)}=\underset{i\in w_{k}}{\sum }\left[{\left(Q_{i}-P_{i}\right)}^{2}+\frac{\lambda }{{\hat{Ґ}}_{G}\left(k\right)}{\left(a_{k}-\gamma_{k}\right)}^{2}\right]$$

where $\gamma_{k}$ is defined as

(21) $$\gamma_{k}=1-\frac{1}{1+e^{ɳ\left(\chi \left(k\right)\right)-\mu_{\chi ,\infty }}}$$

$\mu_{\chi ,\infty }$ is the mean value of all χ(k). ɳ is calculated as $4/\left(\mu_{\chi ,\infty }-\min\left(\chi \left(k\right)\right)\right)$.

${\hat{Ґ}}_{G}\left(k\right)$ is a new edge-aware weighting used to measure the importance of pixel *k* with respect to the whole guidance image. It is defined by using a local variance of 3 × 3 windows and (2ζ1+1)×(2ζ1+1) windows of all pixels by

(22) $${\hat{Ґ}}_{G}\left(k\right)=\frac{1}{N}\overset{N}{\underset{i=1}{\sum }}\frac{\chi \left(k\right)+\varepsilon }{\chi \left(i\right)+\varepsilon }$$

where $\chi \left(k\right)=\sigma_{G,1}\left(k\right)\sigma_{G,\zeta 1}\left(k\right)$. $\zeta_{1}$ is the window size of the filter.

The optimal values of $a_{k}$ and $b_{k}$ are computed by

(23) $$a_{k}=\frac{\mu_{G⊙X,\zeta 1}\left(k\right)-\mu_{G,\zeta 1}\left(k\right)\mu_{X,\zeta 1}\left(k\right)+\frac{\lambda }{{\hat{Ґ}}_{G}\left(k\right)}\gamma_{k}}{\sigma_{G,\zeta 1}^{2}\left(k\right)+\frac{\lambda }{{\hat{Ґ}}_{G}\left(k\right)}}$$

(24) $$b_{k}=\mu_{X,\zeta 1}\left(k\right)-a_{k}\;\mu_{G,\zeta 1}\left(k\right)$$

The final value of $\hat{Q_{i}}$ is calculated by

(25) $$\hat{Q_{i}}={\bar{a}}_{k}G_{i}+{\bar{b}}_{k}$$

where ${\bar{a}}_{k}$ and ${\bar{b}}_{k}$ are the mean values of $a_{k}$ and $b_{k}$ in the window, respectively. ${\bar{a}}_{k}$ and ${\bar{b}}_{k}$ are computed by

(26) $${\bar{a}}_{k}=\frac{1}{\left|w_{\zeta 1}\left(k\right)\right|}\underset{i\in w_{\zeta 1}\left(k\right)}{\sum }a_{k}$$

(27) $${\bar{b}}_{k}=\frac{1}{\left|w_{\zeta 1}\left(k\right)\right|}\underset{i\in w_{\zeta 1}\left(k\right)}{\sum }b_{k}$$

where $\left|w_{\zeta 1}\left(k\right)\right|$ is the cardinality of $w_{\zeta 1}\left(k\right)$.

#### 2.2.6. Refining Weight Maps by Gradient Domain Guided Filter 

Due to these weight maps being noisy and not well aligned with the object boundaries, the proposed approach deploys a gradient domain guided filter to refine the weight maps. The gradient domain guided filter is used at each weight map $W_{n}$ with the corresponding input image $I_{n}$. However, the weigh map $W\_D_{n}$ used $W\_B_{n}$ as the guidance image to improve the $W\_D_{n}$, it is calculated by

(28) $$W\_B_{n}=G_{r1,\varepsilon 1}\left(W_{n},I_{n}\right)$$

(29) $$W\_D_{n}=G_{r2,\;\varepsilon 2}\left(W\_B_{n},I_{n}\right)$$

where r1, ε1 and r2, and ε2 are the parameters of the guided filter. $W\_B_{n}$ and $W\_D_{n}$ are the refined weight maps of the base and detail layers, respectively. Both weight maps $W\_B_{n}$ and $W\_D_{n}$ are deployed using mathematical morphology techniques to remove small holes and unwanted regions in the focus and defocus regions. The morphology techniques are described as bellow,

(30) $$mask\;=W_{n}<threshold\;temp1=imfill\left(mask{,}^{\prime }holes^{\prime }\right)\;temp2=1-temp1\;temp3=imfill\left(temp2{,}^{\prime }holes^{\prime }\right)\;W_{n}\left(refined\right)\;=bwareaopen\left(temp3,threshold\right)$$

Then, the values of the *N* refined weight maps are normalized such that they sum to one at each pixel *k*. Finally, the fused base and detail layer images are calculated and blended to fuse the input images, as given by

(31) $${\bar{B}}_{n}=W\_B_{n}*\;B_{n}$$

(32) $${\bar{D}}_{n}=W\_D_{n}*\;D_{n}$$

(33) $$Fused_{n}={\bar{B}}_{n}+{\bar{D}}_{n}$$

The fast-guided filter is improved by the guided filter proposed by Reference [22]. This algorithm is adopted for reducing the processing of gradient domain guided filter time complexity. Before processing the gradient domain guided filter, the rough transmission map and the guidance image employ nearest the neighbor interpolation down-sampling. After gradient domain guided filter processing, the gradient domain guided filter output image uses bilinear interpolation for up-sampling and obtains the refining transmission map. Using this fast-guided filter, the gradient domain guided filter performs better than the original one. Therefore, the proposed filter was named as the gradient domain fast guided filter.

## 3. Results and Discussion

### 3.1. Multi-Focus Image Fusion

This section describes the comprehensive experiments conducted to evaluate and verify the performance of the proposed approach. The proposed algorithm was developed to fit many types of multi-focus images that are captured by any digital camera or Pi camera. The proposed method was also compared with five multi-focus image fusion techniques: the multi-scale weighted gradient based method (MWGF) [26], the DCT based Laplacian pyramid fusion technique (DCTLP) [27], the image fusion with guided filtering (GFF) [28], the gradient domain-based fusion combined with a pixel-based fusion (GDPB) [29], and the image matting (IM)-based fusion algorithm [30]. The codes of these methods were downloaded and run on the same computer to compare to the proposed method.

The MWGF method is based on the image structure saliency and two scales to solve the fusion problems raised by anisotropic blur and miss-registration. The image structure saliency is used because it reflects the saliency of local edge and corner structures. The large-scale measure is used to reduce the impacts of anisotropic blur and miss-registration on the focused region detection, while the small-scale measure is used to determine the boundaries of the focused regions. The DCTLP presents an image fusion method using Discrete Cosine Transform based Laplacian pyramid in the frequency domain. The higher level of pyramidal decomposition, the better quality of the fused image. The GFF method is based on fusing two-scale layers by using a guided filter-based weighted average method. This method measures pixel saliency and spatial consistency at two scales to construct weight maps for the fusion process. The GDPB method fuses luminance and chrominance channels separately. The luminance channel is fused by using a wavelet-based gradient integration algorithm coupled with a Poisson Solver at each resolution to attenuate the artifacts. The chrominance channels are fused based on a weighted sum of the chrominance channels of the input images. The image mating fusion (IM) method is based on three steps: obtaining the focus information of each source image by morphological filtering, applying an image matting technique to achieve accurate focused regions of each source image, and combining these fused regions to construct the fused image.

All methods used the same input images as the ones applied in the proposed technique. Ten multi-focused image sequences were used in the experiments. Four of them were canola images captured by setting well-focused and manual changing focal length of the Pi camera; the others were selected from the general public datasets used for many image fusion techniques. These general datasets are available in Reference [31,32]. In the first four canola database sets, three of them were artificial multi-focus images obtained by using LunaPic tool [33], one of them was a multi-focus image acquired directly from the Pi camera after cropping the region of interest as described in Section 2.1.

The empirical parameters of the gradient domain fast guided filter and VS metrics were adjusted to obtain the best outputs. The parameters of the gradient domain fast guided filter (see Equation (22)) consisted of a window size filter (ζ1), a small positive constant (ε), subsampling of the fast-guided filter (s), and a dynamic range of input images (L). The parameters of VS maps (Equation (16)), including *α*, *β*, and *γ*, were used to control visual saliency, gradient similarity, and color distortion measures, respectively. These empirical parameters of the gradient domain fast guided filters were experimentally set as s = 4, L = 9, and two pairs of ζ1(1)=4, ε(1)=1.0e−6 and ζ1(2)=4, ε(2)=1.0e−6 for optimizing base and detail weight maps. Other empirical parameters of VS maps were set as *α* = 1, *β* = 0.89, and *γ* = 0.31.

**Figure 3:**
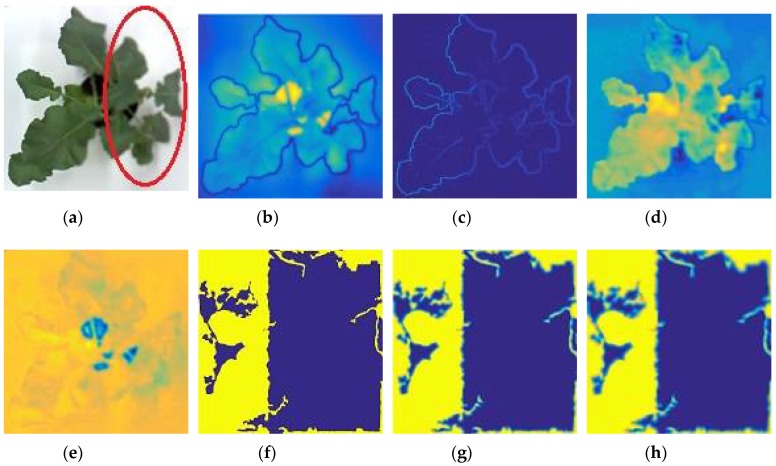
An example of a source image and its saliencies and weight maps: (**a**) A source image; (**b**) visual saliency; (**c**) gradient saliency; (**d**) chrominance color (M); (**e**) chrominance color (N); (**f**) weight maps; (**g**) refined base weight map; (**h**) refined detail weight map.

Surprisingly, when changing these parameters of the VS maps, such as, *α* = 0.31, *β* = 1, and *γ* = 0.31, the fused results had a similar quality to the first parameter settings. It can be thus concluded that to obtain focused regions, both visual saliency and gradient magnitude similarity can be used as the main saliencies. In addition, the chrominance colors (*M* and *N*) also contributed to the quality of the fused results. For example, when increasing the parameters of *M* and *N*, the blurred regions appeared in the fused results. Figure 3 shows the outputs of the proposed algorithm, including visual saliency, gradient magnitude similarity, and chrominance colors. The red oval denotes the defocused region of the input image (Figure 3a).

### 3.2. Comparison with Other Multi-Fusion Methods

In this section, a comprehensive assessment, including both subjective and objective assessment, is used to evaluate the quality of fused images obtained from the proposed and other methods. Subjective assessments are the methods used to evaluate the quality of an image through many factors, including viewing distance, display device, lighting condition, vision ability, etc. However, subjective assessments are expensive and time consuming. Therefore, objective assessments—mathematical models—are designed to predict the quality of an image accurately and automatically.

**Figure 4:**
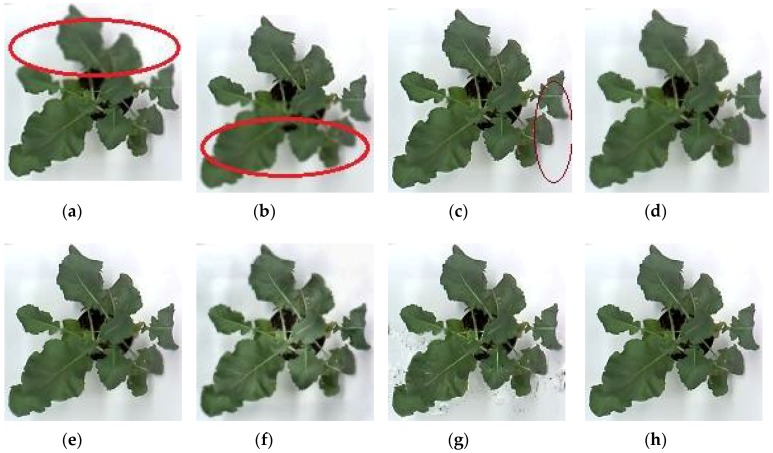
Results: (**a**) Source image 1 of Canola 1; (**b**) Source image 2 of canola 1; (**c**) Reconstructed image using MWGF; (**d**) DCTLP; (**e**) GFF; (**f**) GDPB; (**g**) IM; (**h**) proposed method.

**Figure 5:**
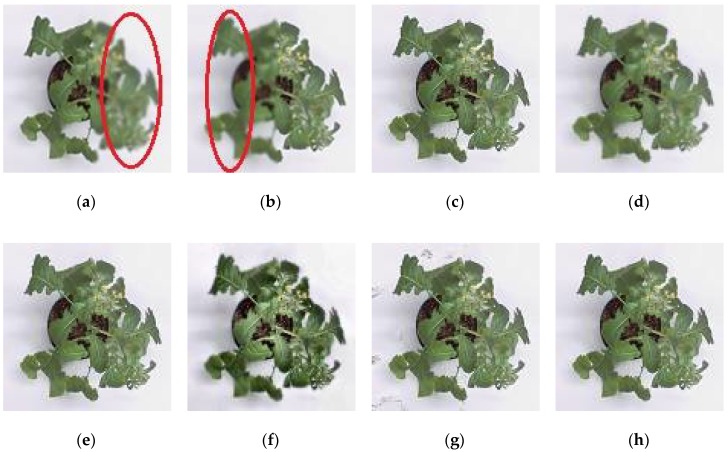
(**a**) Source image 1 of Canola 2; (**b**) Source image 2 of Canola 2; (**c**) MWGF; (**d**) DCTLP; (**e**) GFF; (**f**) GDPB; (**g**) IM; (**h**) proposed method.

**Figure 6:**
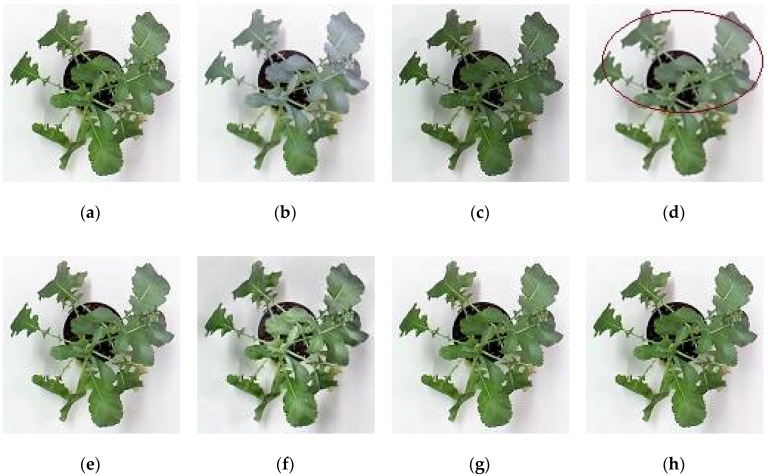
(**a**) Source image 1 of Canola 4; (**b**) Source image 2 of Canola 4; (**c**) MWGF; (**d**) DCTLP; (**e**) GFF; (**f**) GDPB; (**g**) IM; (**h**) proposed method.

**Figure 7:**
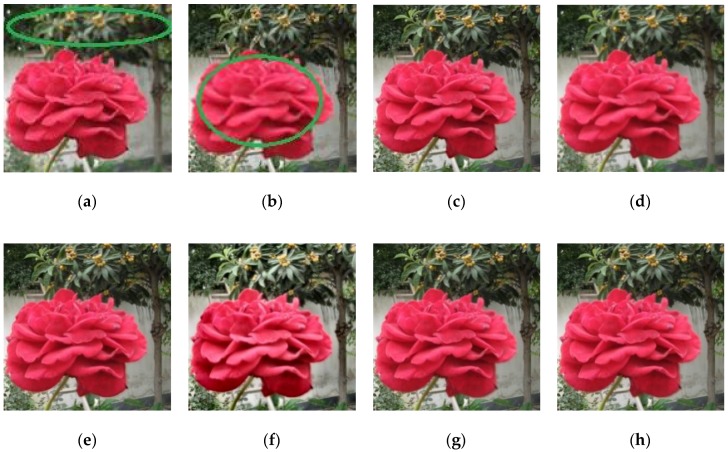
Results: (**a**) Source image 1 of Rose; (**b**) Source image 2 of Rose; (**c**) MWGF; (**d**) DCTLP; (**e**) GFF; (**f**) GDPB; (**g**) IM; (**h**) proposed method.

For subjective or perceptual assessment, the comparisons of these fused images are shown from Figure 4, Figure 5, Figure 6 and Figure 7. The figures show the fused results of the “Canola 1”, “Canola 2”, “Canola 4” and “Rose flower” image sets. In these examples, (a) and (b) are two source multi-focus images, and (c), (d), (e), (f), (g), and (h) are the fused images obtained with the MWGF, DCTLP, GFF, GDPB, IM, and the proposed methods, respectively. In almost all the cases, the MWGF method offers quite good fused images; however, sometimes it fails to deal with the focused regions. For example, the blurred regions remain in the fused image as marked by the red circle in Figure 4c. The DCTLP method offers fused images as good as the MWGF but causes blurring of the fused images in all examples. The IM method also provides quite good results; however, ghost artifacts remain in the fused images, as shown in Figure 4g, Figure 6g, and Figure 7g. Although the fused results of the GFF method reveal good visual effects at first glance, small blurred regions are still remained at the edge regions (the boundary between focused and defocused regions) of the fused results. This blurring of edge regions can be seen in the “Rose flower” fused images in Figure 7e. The fused images of the GDPB method have unnatural colors and too much brightness. The fused results of the GDPB are also suffered from the ghost artifacts on the edge regions and on the boundary between the focused and defocused regions. It can be clearly seen that the proposed algorithm can obtain clearer fused images and better visual quality and contrast than other algorithms due to its combination of the gradient domain fast-guided filter and VS maps. The proposed algorithm offers fused images with fewer block artifacts and blurred edges.

In addition to subjective assessments, an objective assessment without the reference image was also conducted. Three objective metrics, including mutual information (MI) [34], structural similarity (QY) [35], and the edge information-based metric Q(AB/F) [36] were used to evaluate the fusion performance of different multi-focus fusion methods.

The mutual information (MI) measures the amount of information transferred from both source images into the resulting fused image. It is calculated by

(34) $$MI=2\left(\frac{I\left(X,F\right)}{H\left(F\right)+H\left(X\right)}+\frac{I\left(Y,F\right)}{H\left(F\right)+H\left(Y\right)}\right)$$

where I(X,F) is the mutual information of the input image *X* and fused image *F.* I(Y,F) is the mutual information of the input image *Y* and fused image *F*. H(X), H(Y), and H(F) denotes the entropies of the input image *X, Y,* and used image *F*, respectively.

The structural similarity (QY) measures the corresponding regions in the reference original image *x* and the test image *y*. It is defined as

(35) $$Q\left(x,y,f|w\right)=\{\begin{array}{ll} \lambda \left(w\right)SSIM\left(x,f|w\right)+\left(1-\lambda \left(w\right)\right)SSIM\left(y,f|w\right), & for\;SSIM\left(x,y|w\right)\geq 0.75\\ max\left\{SSIM\left(x,f|w\right),\;SSIM\left(y,f|w\right)\right\}, & for\;SSIM\left(x,y|w\right)<0.75 \end{array}$$

where $\lambda \left(w\right)=\frac{s(x|w)}{s\left(x|w\right)+s(y|w)}\;$ is the local weight, and s(x|w) and s(y|w) are the variances of $w_{x}$ and $w_{y}$, respectively.

The edge information-based metric $Q^{AB/F}$ measures the amount of edge information that is transferred from input images to the fused image. For the fusion of source images A and B resulting in a fused image F, gradient strength g(n,m) and orientation α(n,m) are extracted at each pixel (*n*, *m*) from an input image, as given by

(36) $$g_{A}\left(n,m\right)=\sqrt{s_{A}^{x}{\left(n,m\right)}^{2}+s_{A}^{y}{\left(n,m\right)}^{2}}$$

(37) $$\alpha_{A}=tan^{-1}\left(\frac{s_{A}^{y}\left(n,m\right)}{s_{A}^{x}\left(n,m\right)}\right)$$

where $s_{A}^{x}\left(n,m\right)$ and $s_{A}^{y}\left(n,m\right)$ are the output of the horizontal and vertical Sobel templates centered on pixel $p_{A}\left(n,m\right)$ and convolved with the corresponding pixels of input image *A*. The relative strength and orientation values of $G_{}^{AF}\left(n,m\right)$ and $A^{AF}\left(n,m\right)$ of the input image *A* with respect to the fused image *F* are calculated by

(38) $$G^{AF}\left(n,m\right)=\{\begin{array}{ll} \frac{g_{F\left(n,m\right)}\;}{g_{A\left(n,m\right)}} & if\;g_{A\left(n,m\right)}>g_{F\left(n,m\right)}\\ \frac{g_{A\left(n,m\right)}\;}{g_{F\left(n,m\right)}}, & otherwise \end{array}$$

(39) $$A^{AF}\left(n,m\right)=1-\frac{\left|\alpha_{A}\left(n,m\right)-\alpha_{F}\left(n,m\right)\right|}{\pi /2}$$

From these values, the edge strength and orientation values are derived, as given by

(40) $$Q_{g}^{AF}\left(n,m\right)=\frac{{Ґ}_{g}}{1+e^{K_{g}\left(G^{AF}\left(n,m\right)-\sigma_{g}\right)}}$$

(41) $$Q_{\alpha }^{AF}\left(n,m\right)=\frac{{Ґ}_{\alpha }}{1+e^{K_{\alpha }\left(A^{AF}\left(n,m\right)-\sigma_{\alpha }\right)}}$$

$Q_{g}^{AF}\left(n,m\right)$ and $Q_{\alpha }^{AF}\left(n,m\right)$ model information loss between the input image *A* and the fused image *F*. The constants ${Ґ}_{g}$, $K_{g}$, $\sigma_{g}$ and ${Ґ}_{\alpha }$, $K_{\alpha },\;\sigma_{\alpha }$ determine the exact shape of the sigmoid functions used to form the edge strength and orientation preservation values (Equation 40 and Equation 41). Edge information preservation values are formed by

(42) $$Q^{AF}\left(n,m\right)=\;Q_{g}^{AF}\left(n,m\right)Q_{\alpha }^{AF}\left(n,m\right)$$

with $0\leq Q^{AF}\left(n,m\right)\leq 1$. The higher value of $Q^{AF}\left(n,m\right)$, the less loss of information of the fused image.

The fusion performance $Q^{\mathrm{AB}/F}$ is evaluated as a sum of local information preservation estimates between each of the input images and fused image, it is defined as

(43) $$Q^{AB/F}=\frac{\sum_{n=1}^{N}\sum_{m=1}^{M}Q^{AF}\left(n,m\right)w^{A}\left(n,m\right)+Q^{BF}\left(n,m\right)w^{B}\left(n,m\right))}{\sum_{j=1}^{N}\sum_{j=1}^{M}(w^{A}\left(i,j\right)+w^{B}\left(i,j\right))}$$

where $Q^{AF}\left(n,m\right)$ and $Q^{BF}\left(n,m\right)$ are edge information preservation values, weighted by $w^{A}\left(n,m\right)$ and $w^{B}\left(n,m\right)$, respectively.

Table 1 illustrates the quantitative assessment values of five different multi-focus fusion methods and the proposed method. The larger the value of these metrics, the better image quality is. The values shown in bold represent the highest performance. From Table 1, it can be seen that the proposed method produces the highest quality scores for all three objectives metrics except for QY with “Canola 2” datasets and QAB/F with “Book” (extra images were also run to test the performance). These largest quality scores imply that the proposed method performed well, stably, and reliably. Overall, it can be concluded that the proposed method reveals the competitive performance when compared with previous multi-focus fusion methods both in visual perception and objective metrics. Table 2 describes the ranking of the proposed method with others based on the quality of fused images. The performance (including quality of the images and the processing time) is scaled from 1 to 6. The results show the outperformance of the proposed technique with other techniques previously published.

## 4. Summary and Conclusions

To improve the description and quality images, especially images acquired from the digital camera or the Pi camera for canola phenotyping, an image fusion method is necessary. A new multi-focus image fusion method was proposed with the combination of the VS maps and gradient domain fast guided filters. In the proposed algorithm, the VS maps were first deployed to obtain visual saliency, gradient magnitude similarity saliency, and chrominance saliency (or color distortions), then the initial weight map was constructed with a mix of three metrics. Next, the final decision weight maps were obtained by optimizing the initial weight map with a gradient domain fast guided filter at two components. Finally, the fused results were retrieved by the combination of two-component weight maps and two-component source images that present large-scale and small-scale variations in intensity. The proposed method was compared with five proper representative fusion methods both in subjective and objective evaluations. Based on the experiment’s results, the proposed fusion method presents a competitive performance with or outperforms some state-of-the-art methods based on the VS maps measure and gradient domain fast guided filter. The proposed method can use digital images which are captured by either a high-end or low-end camera, especially the low-cost Pi camera. This fusion method can be used to improve the images for trait identification in phenotyping of canola or other species.

On the other hand, some limitations of the proposed multi-focus image fusion, such as small-blurred regions in the boundaries between the focused and defocused regions and computational cost, are worthwhile to investigate. Morphological techniques and optimizing the multi-focus fusion algorithm are also recommended for further study.

Furthermore, 3D modeling from enhancing depth images and image fusion techniques should be investigated. The proposed fusion technique can be implemented in the phenotyping system which has multiple sensors, such as thermal, LiDAR, or high-resolution sensors to acquire multi-dimensional images to improve the quality or resolution of the 2D and 3D images. The proposed system and fusion techniques can be applied in plant phenotyping, remote sensing, robotics, surveillance, and medical applications.

## Figures and Tables

**Table 1:** Comparison of the proposed method with other methods.

Index	Source Images	Methods					
		MWGF	DCTLP	IM	GFF	GDPB	Proposed Algorithm
**QMI**	Canola 1	1.224	1.042	1.124	1.190	0.656	1.288
	Canola 2	1.220	0.946	1.164	1.147	0.611	1.230
	Canola 3	1.165	0.981	1.043	1.148	0.573	1.212
	Canola 4	1.320	0.943	1.400	1.060	0.570	1.400
	Doll	0.664	0.918	0.881	0.310	0.732	1.011
	Rose	1.049	1.133	1.002	0.440	0.736	1.147
	Jug	1.065	1.085	0.974	0.347	0.742	1.094
	Diver	1.168	1.207	1.190	0.515	0.910	1.210
	Book	0.957	1.188	1.152	0.487	0.900	1.234
	Notebook	1.118	1.181	1.141	0.463	0.745	1.190
**QY**	Canola 1	0.958	0.851	0.812	0.948	0.755	0.970
	Canola 2	0.981	0.859	0.901	0.967	0.762	0.980
	Canola 3	0.961	0.856	0.752	0.955	0.737	0.970
	Canola 4	0.777	0.799	0.980	0.913	0.700	0.980
	Doll	0.902	0.950	0.960	0.800	0.872	0.980
	Rose	0.973	0.979	0.973	0.829	0.901	0.980
	Jug	0.995	0.990	0.970	0.970	0.779	0.995
	Diver	0.975	0.971	0.976	0.744	0.918	0.976
	Book	0.952	0.956	0.959	0.647	0.850	0.977
	Notebook	0.987	0.983	0.991	0.844	0.816	0.992
**QAB/F**	Canola 1	0.958	0.885	0.938	0.930	0.883	0.974
	Canola 2	0.987	0.987	0.981	0.987	0.987	0.987
	Canola 3	0.955	0.621	0.937	0.841	0.607	0.970
	Canola 4	0.906	0.492	0.915	0.529	0.481	0.915
	Doll	0.987	0.986	0.987	0.986	0.987	0.987
	Rose	0.987	0.987	0.987	0.986	0.987	0.987
	Jug	0.987	0.987	0.987	0.986	0.987	0.987
	Diver	0.986	0.986	0.986	0.986	0.986	0.986
	Book	0.984	0.980	0.984	0.984	0.983	0.984
	Notebook	0.986	0.987	0.987	0.986	0.987	0.987

**Table 2:** Ranking the performance of fused images of the proposed method with other methods based on the results from Table 1.

Source Images	Methods					
	MWGF	DCTLP	IM	GFF	GDPB	Proposed Algorithm
Canola 1	2	5	4	3	6	1
Canola 2	2	5	4	3	6	1
Canola 3	2	5	4	3	6	1
Canola 4	2	4	1	3	5	1
Doll	5	2	3	6	4	1
Rose	3	2	4	5	6	1
Jug	3	2	4	6	5	1
Diver	4	2	3	6	5	1
Book	4	2	3	6	5	1
Notebook	4	3	2	6	5	1
